# Supplementary material for: “Best Paper” awards lack transparency, inclusivity, and support for Open Science
Source: PLoS Biol. 2024 Jul 23;22(7):e3002715. doi: 10.1371/journal.pbio.3002715 (PMC11265724; doi:10.1371/journal.pbio.3002715)
Supplement: Attachment — Submitted filename: review-v1.pdf [file pbio.3002715.s003.pdf]

Dear Editor,

Thank you for the opportunity to review this manuscript.

Overall, this paper makes an interesting and, to my knowledge, novel contribution to meta-research. Its focus on best paper awards brings attention to an incentive that has received less attention in the academic literature and ongoing research reform, and could serve to spark further discussion and change.

Furthermore, the authors "practices what they preach" with regards to open science: their study is pre-registered with a public protocol, and code and data is made available. Please note that due to time constraints, I could not review the protocol, code, or data in detail.

I had troubles interacting with the wonderfully detailed supplement and would suggest formatting changes: The PDF appears to be a printed Quarto-generated HTML, which is great for reproducibility; perhaps the HTML webpage (e.g., hosted on a GitHub Pages) could be linked within the PDF for better native navigation. The PDF pages are far larger than A4 and hard to use; I would suggest making these A4 and forcing page breaks for legibility. Additionally, the hyperlinks do not work, and with so many sections and so few pages, this made it a challenge to navigate; I imagine this is due to the peer review software and the final supplement would be "clickable." Also, it would be helpful to have numbered/lettered sections of the supplement, if journal policy allows, as it is extensive, and I found myself searching for the correct section. Additionally, due to time constraints, I was not able to review the supplement in detail.

The authors argue that best paper awards fall short in three interlinked areas: award criteria and details are not sufficiently explicit and clear to applicants and other scientists ("transparency"); awards make insufficient efforts to be inclusive of a broader range of scientists ("inclusivity"); awards give insufficient weight to responsible research practices highlighted in ongoing research reforms ("open science"). The first two ("transparency" and "inclusivity") regard how the award description is written or "award characteristics," per the authors; whereas "open science" regards the research itself. However, the biggest challenge I saw was the lack of clear delineation and connections between these dimensions; I felt the terminology somewhat was insuffiently defined and used too interchangeably, within the three areas, as well as with additional terminology (e.g., sometimes "inclusivity" and sometimes "diversity", though these are separate although related concepts). I believe that a careful pass of the language with some additional or re-writing as needed could help resolve these challenges. Below are some specific instances to help clarify what I mean:

- Title includes "transparency, ***inclusivity***, and support for Open Science," while abstract includes "transparency, ***diversity***, and openness in science" (emphasis added)
- As "research ***transparency***" is a term often used within ***open science*** (as on **lines 217, 272, 300**, etc.), it wasn't always clear whether "transparency" refered to the award (i.e., whether eligibility info is explicit) or the awarded research (i.e., whether the research is transparent about what was done; i.e., open science). Personally it would be clearest using another term, but as Lagisz and colleagues have previously published on this topic with this terminology, I would understand

their desire for consistency, and, in that case, the use of the terms should be made clearer.

- In the discussion, the paragraph from **lines 292 - 317** starts with "Do Best Papers awards follow ***equitable*** practices or the path of least resistance?" (emphasis added) and then proceeds to discuss impact metrics and open science. However, is the issue at hand primarily one of inequity (open science practices may not always foster and may even extend inequity per Ross-Hellauer 2022 https://doi.org/10.1038/d41586-022-00724-0), or rather that the awards may not be comprehensively evaluating the "bestness" of papers by using simplistic measures (similar to what is argued in research reform)?

In their discussion, the authors argue that the demonstrated shortcomings of current best paper awards merit changes to the design of awards, and they provide concrete suggestions. I would be curious to learn how the authors think their findings might be taken into consideration with ongoing internation research evaluation reform (e.g., CoARA) and believe this would merit being addressed in the discussion beyond the brief reference on **line 299**.

Below are comments regarding specific lines:

73-74: The authors mention "global calls," which I then expected to be elaborated in the paper, but I found this just in a brief reference on **line 299**. Please make this link clearer.

87-98: The claim about the Matthew Effect and historically disadvantages and marginalized groups would be stronger with a reference, if available.

90: Do the authors mean "Studying awards" instead of "Awards"?

92: What "related institutions"? Those giving the awards, e.g., "granting institutions"?

103: What do the authors mean by "the ones most valued"?

115: I would suggest more specific language than "lack of transparency," as I first read this as open-science-related.

121: "Prizes" terminology is first introduced here. I had to re-reread since I first thought this was different than awards and instead were what the authors later call "perks." Perhaps establish these as synonyms, possibly even earlier.

125: What do the authors mean by "diversity of work"?

134-137: While I intuitively agree with this claim, I would suggest either citing or soften the language.

138-139: Are previous studies revealing opaque best paper award criteria limited to reference 33 in computer science? Please reword to clarify.

146: What does it mean for an award to "consider equity and inclusion"? Echoing an overarching comment above, do the authors mean whether the research itself is equitable and inclusive, or access to the award? Also, why these specific terms?

150: I was surprised to see that a main aim was to evaluate "gender and affiliation country," given the broader scope presented in the introduction. The authors make this feasibility-based choice clearer later on, but it would have helped me as a reader to already hint at this in the introduction.

156: Thanks for this great workflow chart! One question towards the bottom: what is "less relevant SA"? Please specificy how this was determined, e.g. in a figure caption.

157: Thanks for sharing the OSF link (https://osf.io/8mxpq/) with your protocol. I am confused about the versioning with two files called "BP_awards_protocol_final.pdf": https://osf.io/93256 created & modified December 26, 2022, and https://osf.io/vresm created November 12, 2022 & modified September 17, 2023. It seems the pdf's are both the same and dated 25 December 2022 and one could be removed for clarity.

158: I believe you searched the top-SCImago-ranked 100 journals OR until 10 awards were found. Please integrate this into the brief methods summary, as it would have helped to understand the subsequent numbers.

163: What do the authors mean by "characteristics *representing*..." (emphasis added)?

165: The authors detail how gender was "coded" in the methods and limitations (**lines 375-377**). I would suggest to soften the language here to "we inferred the winning author's gender."

166-167: I was confused about the "eligible awards," which though explained later, could be briefly made clearer here.

177-179: Please clarify why the percentages add to over 100.

179-180: I am curious what sectors the "other organizations" come from. From the parenthetical, it seems limited to Elsevier and Wiley. The sentence could be clarified.

182-204: I found this section challenging to follow. Regarding the header "Awards - equity and transparency," I am unsure why the term "equity" is used. As for "transparency," based on it's earlier introduction, I understand "transparency" to refer to the clarity of award details, which then also covers the subsequent section on assessment criteria. I saw two areas addressed: first, eligibility criteria which includes single/team, career stage, historically underrepresented and marginalized scholars; second, award information which includes assessors, conflicts of interest, (self-)nomination, feedback, contact details. I recognize this section is hard to structure; splitting it up might help.

196: Do the authors mean "reported" rather than "informed"?

200: Is "process transparency" used to indicate only whether information about nominated articles is provided? This is what I understand from the main text (only one award) and Fig 3A, but Fig S9 has more that 1 "yes" (arts and humanities; nursing). Please clarify this number. If "process transparency" does mean this single practice, the authors might consider removing this extra term and simply specifying the practice for clarity.

208: Might the authors communicate their result with less judgment by removing "not totally opaque," i.e. "assessment criteria that were not restricted to statements..."?

220: It would be interesting to know generally what type of awardee data was available, if any, beyond name (and in part affiliation). For example, was gender ever provided? Affiliation at time of the award?

228: Was affiliation information retrieved manually or using code?

Fig 5: This data may be better suited to another type of plot, especially given the difference in number of years for the current decade. As I am not a data visualization expert, I unfortunately cannot suggest further. Figs 5C & D have too many layers and colors in the stacked bar chart, making it hard to read. Perhaps a map-based density chart would work.

295: What are "impact awards" and why could they not be included?

296-297: The authors might strengthen their critical argument against impact indices with a reference.

309: Why is "increasingly" in parentheses?

315-316: That best paper awards "contribute to an invaluable sense of achievement and belonging, especially for minority and underrepresented researchers" is a strong argument and should be cited or made clear that this claim is made on the basis of the presented research (which I don't believe is the case).

317: As I wrote above, this paragraph does not seem to focus on "equitability and inclusiveness." I think this important argument and reflection could be strengthened by restructuring this paragraph and the subsequent one.

333-346: I applaud the authors' attempt to provide more actionable recommendations based on their research, and appreciated their used of an established framework (thank you for introducing me to the SPACE rubric). However, as this framework is extensive and rather complex, I wonder if it could be accompanied by a concrete example (e.g., in a "Box" in the article) of a specific institution granting a best paper award, perhaps one that is further along in the reform. If feasible, this would help not only academic readers, but also journal leadership looking to apply this framework to reform their journal's awards. A few comments within the table:

- **Standards for scholarship**: Perhaps list a few "buzzwords" so that readers looking at only this table can follow. The last two bullets seem to go beyond the "foundation" column, and could be reworded to focus on the hard work of established shared understanding.
- **Process mechanics and policies**: Again "ensure" sounds like it goes beyond "foundation." What is meant by "the narrow circle"?

349: Recommendations to researchers seem to revolve around asking/communicating with the awarding body; perhaps this could be more informative in the paragraph's topic sentence that "straightforward."

377-378: The authors mention one additional dimension of researcher diversity which they considered and decided not to include. If other dimensions were considered, such as those in **lines 396-397**, please reference them. If not, please elaborate on how the dimensions were selected, first, for consideration, and, second, for analysis.

380-382: This claim would be strengthened by a reference.

440-441: How did you select "awards that carry the most prestige"? Is this by SCImago ranking?

447-449: On my first reading, it was unclear whether the "10 potentially relevant awards" were per journal or field. This might be clarified by rephrasing the sentence to leading with "We stopped screening each Subject Area...".

468-469: Are the award description pdfs made available? I had trouble finding them in https://zenodo.org/doi/10.5281/zenodo.10056492.

507-509: Kudos to the authors for the open data and code. The OSF at https://osf.io/yzr7a/ seems to be private and should be made accessible. The GitHub repo has an extensive readme, which is helpful for orientation. However, I imagine I would struggle to reuse the data and would suggest adding a data readme, list of datafiles/csvs, and a codebook. Recognizing that this project produced many intermediate files, I suggest more detailed documentation at least of the final version of the data used for the analysis and claims presented in the paper; this database of awards and winners, complimented by the research team's manual coding, could be useful to future potential award applicants and meta-researchers alike. Please note that I recognise the time-consuming nature and pain of data documentation and thank the authors in advance for this extra effort for the sake of reusability.

To reiterate, this paper makes a valuable addition to the meta-research literature, and I believe the outstanding issues could be addressed in a revision. I ask the editor to please note the limitations of my review due to time constraints; I could not extensively review the supplement, protocol, code, or data.

Best regards,

Maia Salholz-Hillel
