# Supplementary material for: “Best Paper” awards lack transparency, inclusivity, and support for Open Science
Source: PLoS Biol. 2024 Jul 23;22(7):e3002715. doi: 10.1371/journal.pbio.3002715 (PMC11265724; doi:10.1371/journal.pbio.3002715)
Supplement: Attachment — Submitted filename: Review for PLOSBiology March 2024-Gerben ter Riet.docx [file pbio.3002715.s002.docx]

I enjoyed reviewing this manuscript. In my opinion it constitutes important and original work, clear objectives and using good methodologies, while drawing defensible and important conclusions that are supported by the data.

The paper shows that in a sample of 222 Best Paper awards across 27 disciplines a lot can be gained in terms of transparency of the organizational processes around these awards (opaque award criteria) and in terms of the required scientific features deemed important for submissions. For example, highlighting features such as outstanding and original, while rarely emphasizing e.g. the reproducibility or transparency of the work.

I am slightly less convinced by the equity part (EDI) of the paper (“to 150 evaluate potential gender and affiliation country disparities in the lists of past awardees.”), which deals mainly with gender and country. I think this is because it is hard to exactly define from which point on some proportion proves that the mechanisms behind the awards are biased. For example, does the decrease in the proportion of award winning US papers after 2010 prove that the award processes were (more) biased between 2001 and 2010? In addition, the authors – for understandable reasons – refrained from assessing the quality (if that can be defined) of the award winning papers, hindering insights in whether the relative quality of US-based winners decreased after 2010 and may have caused the smaller proportion after 2010. The question of gender bias in research and science is complex and the authors may seriously consider to enrich their discussion by extending it to discussions around gender bias in grant application (e.g. <https://bmjopen.bmj.com/content/10/8/e035058> ). After, all obtaining grants for many researchers is the foundation for writing research papers at all.

I agree with the authors that Best Paper awards have an important signaling function to (young) researchers on what good research is about. The lack of transparency and the emphasis on spectacular outcomes rather than reliable methods (process) is worrying. The current manuscript builds on earlier work by these authors (they also indicate that) in the field of ecology and evolution and extends it across 27 disciplines.

The manuscript is well written and the figures are informative (I have 1 minor query in figure 1a: what does the vertical line of dots indicate? Add that to the legend). Importantly, the authors themselves preregistering their protocol. I was unable to access their sharing data and code (via osf.io). The paper would gain additional strength if data and code were shared.

In their **Abstract**, the authors write that ‘Instead, such awards increasingly rely on article-level impact metrics.’ Since their work is based on a cross-sectional study, I wondered if they can truly claim the ‘increasingly’, which implies longitudinal data.

In their **Introduction** the authors loosely build an argument from an emphasis on striking results (rather than robust methods) and privileged marginalized groups. Although I understand their message, the line of argument from striking results to disadvantaged groups might be strengthened.

The **Methods** section is clear and informative. I wonder if the paper may shed some light (possibly hindered by small numbers) on the question whether awards endorsing EDI and/or open science features are more likely to be transparent in their award criteria.

In the **Results** section, how do the authors explain the rise in the number of Best Paper awards after 2010?

The **Conclusions** section reads more as a manifesto and although I am personally sympathetic to what they say, I wonder if these really can be counted as conclusions. A suggestion for the section title might be ‘Further action’. The sentence ‘Rectifying such biases is our collective responsibility.’ assumes that there are biased, and there may be, but the data in my opinion do not really support this assumption. Extending the discussion section to the literature on biased in grant application procedures may be valuable.

Gerben ter Riet, MD PhD

Amsterdam, The Netherlands
