# Supplementary material for: “Best Paper” awards lack transparency, inclusivity, and support for Open Science
Source: PLoS Biol. 2024 Jul 23;22(7):e3002715. doi: 10.1371/journal.pbio.3002715 (PMC11265724; doi:10.1371/journal.pbio.3002715)
Supplement: Attachment — Submitted filename: RESPONSES.docx [file pbio.3002715.s004.docx]

RESPONSES TO REVIEWERS

REVIEWERS' COMMENTS:

Reviewer #1:

[identifies himself as Gerben ter Riet; see attachment for formatted version]

I enjoyed reviewing this manuscript. In my opinion it constitutes important and original work, clear objectives and using good methodologies, while drawing defensible and important conclusions that are supported by the data.

RESPONSE 1:

Thank you for taking the time to read our manuscript carefully and for recognising the importance of our work.

The paper shows that in a sample of 222 Best Paper awards across 27 disciplines a lot can be gained in terms of transparency of the organizational processes around these awards (opaque award criteria) and in terms of the required scientific features deemed important for submissions. For example, highlighting features such as outstanding and original, while rarely emphasizing e.g., the reproducibility or transparency of the work.

RESPONSE 2:

Thank you for this accurate summary of our main findings and highlighting the potential impact of our work.

I am slightly less convinced by the equity part (EDI) of the paper ("to 150 evaluate potential gender and affiliation country disparities in the lists of past awardees."), which deals mainly with gender and country. I think this is because it is hard to exactly define from which point on some proportion proves that the mechanisms behind the awards are biased. For example, does the decrease in the proportion of award winning US papers after 2010 prove that the award processes were (more) biased between 2001 and 2010? In addition, the authors - for understandable reasons - refrained from assessing the quality (if that can be defined) of the award winning papers, hindering insights in whether the relative quality of US-based winners decreased after 2010 and may have caused the smaller proportion after 2010. The question of gender bias in research and science is complex and the authors may seriously consider to enrich their discussion by extending it to discussions around gender bias in grant application (e.g. <https://bmjopen.bmj.com/content/10/8/e035058>). After, all obtaining grants for many researchers is the foundation for writing research papers at all.

RESPONSE 3:

This is a very insightful and valuable comment. We agree that it is hard to infer the mechanisms from proportions of winners falling into certain categories without having any specific baseline information. Thus, in our work we focus on describing observed patterns in the data rather than stating that the award mechanisms are biased. Further, our recommendations for the awarding bodies are to conduct their own internal assessments of biases and mechanisms that may contribute to such biases, for example:

New lines 542-545: “Fourth, when assigning gender to the past winners, in 58% of cases we only had a person’s first name as the basis of our coding (we also relied on pronouns and photos when available). We acknowledge that the person’s publishing name (and also officially used images or pronouns) may not accurately reflect the person's gender self-identification [59].”

Table 1: “Work on diagnosing the nature of inequities that exist in the current (or traditional) policies and procedures.”

Thank you for suggesting extending our discussion to gender biases in grant applications. We incorporated this as follows:

New lines 459-460: “Numerous studies reported gender biases in expert evaluation of grant proposals, with varying time-trends (see [55]).”

I agree with the authors that Best Paper awards have an important signaling function to (young) researchers on what good research is about. The lack of transparency and the emphasis on spectacular outcomes rather than reliable methods (process) is worrying. The current manuscript builds on earlier work by these authors (they also indicate that) in the field of ecology and evolution and extends it across 27 disciplines.

RESPONSE 4:

Thank you for your positive feedback.

The manuscript is well written and the figures are informative (I have 1 minor query in figure 1a: what does the vertical line of dots indicate? Add that to the legend). Importantly, the authors themselves preregistering their protocol. I was unable to access their sharing data and code (via [osf.io](http://osf.io/) ). The paper would gain additional strength if data and code were shared.

RESPONSE 5:

We apologise for not describing the vertical dotted line in figure 1A. We now added a description to the figure legend:

New lines 888-889: “*Vertical dotted line in panel A indicates a cut-off of 10 awards per SCImago Subject Area.”*

We also apologise for the problems with accessing documents stored online on OSF. We have changed the status of the repository to public access. The data and code are available on GitHub and archived on Zenodo under the links that are provided in our manuscript at the end of the Methods section.

New lines 685-687: “All data and code are openly available through a dedicated OSF project (https://osf.io/yzr7a/), Zenodo (<https://doi.org/10.5281/zenodo.11215401>), and GitHub repository (https://github.com/mlagisz/survey_best_paper_awards).”

In their Abstract, the authors write that 'Instead, such awards increasingly rely on article-level impact metrics.' Since their work is based on a cross-sectional study, I wondered if they can truly claim the 'increasingly', which implies longitudinal data.

RESPONSE 6:

Thank you for your valuable comment. Indeed, this is a cross-sectional study, but we also collected data that approximates when each award has been established (the earliest year of the listed past winners). We assumed that award criteria would not change much since the establishment of the award and used the estimated award age as a predictor of award characteristics. We have tempered our language and made clearer the sentence in question to state the exact number, and the fact that we are talking about awards that were recently established, rather than implying using longitudinal data:

New lines 70-71: “Instead, 10% of awards, especially the recently established ones, tend to use article-level impact metrics.”

In their Introduction the authors loosely build an argument from an emphasis on striking results (rather than robust methods) and privileged marginalized groups. Although I understand their message, the line of argument from striking results to disadvantaged groups might be strengthened.

RESPONSE 7:

We apologise for any confusion that arose when reading this section. Following suggestions from all reviewers, we have made numerous changes to this section, including rewriting some of the statements and adding supporting references. We believe that these changes have streamlined and strengthened our Introduction section.

The Methods section is clear and informative. I wonder if the paper may shed some light (possibly hindered by small numbers) on the question whether awards endorsing EDI and/or open science features are more likely to be transparent in their award criteria.

RESPONSE 8:

This is a great question. Unfortunately, as the reviewer already highlighted, small numbers preclude such analyses. We found only two awards that endorsed EDI and one that explicitly valued Open Science practices. There was no overlap between these awards. It is currently not possible to conduct a robust analysis testing this interesting prediction.

In the Results section, how do the authors explain the rise in the number of Best Paper awards after 2010?

RESPONSE 9:

It appears to be a more general trend according to the work of Joel Best, which we now note and cite in our Discussion section as follows:

New lines 318-319: “Our results indicate that Best Paper awards are relevant to any career stage and discipline, and are growing in number (prize proliferation has been already noted in 2008 [44]).”

The Conclusions section reads more as a manifesto and although I am personally sympathetic to what they say, I wonder if these really can be counted as conclusions. A suggestion for the section title might be 'Further action'. The sentence 'Rectifying such biases is our collective responsibility.' assumes that there are biased, and there may be, but the data in my opinion do not really support this assumption. Extending the discussion section to the literature on biased in grant application procedures may be valuable.

RESPONSE 10:

Thank you for your suggestions. We changed the section title to “Conclusions and further action”, which we hope will accommodate the convention of having the last section titled “Conclusions” and also its role of calling for a rehaul of scientific awards. The sentence 'Rectifying such biases is our collective responsibility.' should be read in the context of the preceding sentence that asks for evaluation of “potential biases”, so if no biases were found no rectifying would be needed. We now added the word “potential” to the sentence in question:

New lines 594: “Rectifying such potential biases is our collective responsibility.”

We now also touch upon gender biases in grant applications as follows:

New lines 459-460: “Numerous studies reported gender biases in expert evaluation of grant proposals, with varying time-trends (see [55]).”

Gerben ter Riet, MD PhD

Amsterdam, The Netherlands

## **Reviewer #2:**

[identifies herself as Maia Salholz-Hillel; see attachment for formatted version]

Dear Editor,

Thank you for the opportunity to review this manuscript.

Overall, this paper makes an interesting and, to my knowledge, novel contribution to meta-research. Its focus on best paper awards brings attention to an incentive that has received less attention in the academic literature and ongoing research reform, and could serve to spark further discussion and change.

RESPONSE 11:

Thank you for taking the time to read our manuscript carefully and for recognising the relevance of our work to the research community.

Furthermore, the authors "practices what they preach" with regards to open science: their study is pre-registered with a public protocol, and code and data is made available. Please note that due to time constraints, I could not review the protocol, code, or data in detail.

RESPONSE 12:

Thank you for your positive and valuable feedback on our study.

I had troubles interacting with the wonderfully detailed supplement and would suggest formatting changes: The PDF appears to be a printed Quarto-generated HTML, which is great for reproducibility; perhaps the HTML webpage (e.g., hosted on a GitHub Pages) could be linked within the PDF for better native navigation. The PDF pages are far larger than A4 and hard to use; I would suggest making these A4 and forcing page breaks for legibility. Additionally, the hyperlinks do not work, and with so many sections and so few pages, this made it a challenge to navigate; I imagine this is due to the peer review software and the final supplement would be "clickable." Also, it would be helpful to have numbered/lettered sections of the supplement, if journal policy allows, as it is extensive, and I found myself searching for the correct section. Additionally, due to time constraints, I was not able to review the supplement in detail.

RESPONSE 13:

We apologise for the difficulties navigating our supplementary file. The reviewer has correctly stated that it is a printed markdown-generated html, which was further disjointed by the submission system. Our intention was to have the final document available as an interactive html file both alongside the published manuscript and as a GitHub-hosted page. We have made the necessary changes and further improvements to the structure and content of the supplement to make it ready for the final submission and online publication.

The authors argue that best paper awards fall short in three interlinked areas: award criteria and details are not sufficiently explicit and clear to applicants and other scientists ("transparency"); awards make insufficient efforts to be inclusive of a broader range of scientists ("inclusivity"); awards give insufficient weight to responsible research practices highlighted in ongoing research reforms ("open science"). The first two ("transparency" and "inclusivity") regard how the award description is written or "award characteristics," per the authors; whereas "open science" regards the research itself. However, the biggest challenge I saw was the lack of clear delineation and connections between these dimensions; I felt the terminology somewhat was insuffiently defined and used too interchangeably, within the three areas, as well as with additional terminology (e.g., sometimes "inclusivity" and sometimes "diversity", though these are separate although related concepts). I believe that a careful pass of the language with some additional or re-writing as needed could help resolve these challenges. Below are some specific instances to help clarify what I mean:

· Title includes "transparency, ***inclusivity***, and support for Open Science," while abstract includes "transparency, ***diversity***, and openness in science" (emphasis added)

RESPONSE 14:

Thank you for pointing out these inconsistencies and the specific suggestions on where to make improvements. We carefully edited the whole manuscript and made changes in line with these suggestions, including making the abstract consistent with the title:

New lines 64: “But do awards incentivise greater transparency, inclusivity, and openness in science?”

New lines 74-76: “We provide concrete and implementable recommendations for scientific awards to improve the scientific recognition system and incentives for better scientific practice.”

New lines 289-294: “Practices relevant to Open Science (such as transparency, reproducibility, and robustness) were mentioned only in the criteria of a single award (0.5%; Review Of Research Award by the American Educational Research Association; Fig S17) explicitly emphasizing “transparency of the methods”. Additional text mining of the award descriptions revealed that words such as “best”, “outstanding”, “original”, and “impactful” frequently appear in award descriptions, but words related to reproducibility and robustness are rarely (if ever) mentioned (Fig 4).”

New lines 902-903: “***Fig 3. Summary of characteristics of included awards, derived from award descriptions.*** *(A) General characteristics related to accessibility, inclusivity and transparency.”*

· As "research ***transparency***" is a term often used within ***open science*** (as on **lines 217, 272, 300**, etc.), it wasn't always clear whether "transparency" refered to the award (i.e., whether eligibility info is explicit) or the awarded research (i.e., whether the research is transparent about what was done; i.e., open science). Personally it would be clearest using another term, but as Lagisz and colleagues have previously published on this topic with this terminology, I would understand their desire for consistency, and, in that case, the use of the terms should be made clearer.

RESPONSE 15:

Thank you for spotting this inconsistency which may confuse readers. To reduce confusion, we now use the term “transparency” in relation to award descriptions, rather than research works:

New lines 292-295: “Additional text mining of the award descriptions revealed that words such as “best”, “outstanding”, “original”, and “impactful” frequently appear in award descriptions, but words related to reproducibility and robustness are rarely (if ever) mentioned (Fig 4)..”

New lines 3263-384: “Words related to comprehensive reporting, rigor, and reliability were almost non-existent, indicating that related qualities may not be considered when evaluating published works.”

New lines 424-426: “Among our findings, the lack of mentions (in all but one case) of the importance of adherence to Open Science practices shows clearly that such practices are not valued (or at least not articulated publicly as valuable or required).”

· In the discussion, the paragraph from **lines 292 - 317** starts with "Do Best Papers awards follow ***equitable*** practices or the path of least resistance?" (emphasis added) and then proceeds to discuss impact metrics and open science. However, is the issue at hand primarily one of inequity (open science practices may not always foster and may even extend inequity per Ross-Hellauer 2022 <https://doi.org/10.1038/d41586-022-00724-0>), or rather that the awards may not be comprehensively evaluating the "bestness" of papers by using simplistic measures (similar to what is argued in research reform)?

RESPONSE 16:

We have rephrased the sentence following this valuable comment:

New lines 403-404: “Do Best Papers awards recognise research quality or follow the path of least resistance?”

In their discussion, the authors argue that the demonstrated shortcomings of current best paper awards merit changes to the design of awards, and they provide concrete suggestions. I would be curious to learn how the authors think their findings might be taken into consideration with ongoing internation research evaluation reform (e.g., CoARA) and believe this would merit being addressed in the discussion beyond the brief reference on **line 299**.

RESPONSE 17:

Thank you for your constructive feedback. We now explicitly mention DORA and CoARA initiatives in our discussion:

New lines 361-364: “Notably, initiatives such as the San Francisco Declaration on Research Assessment (DORA) and Coalition for Advancing Research Assessment (CoARA) champion moving away from simplistic quantitative indicators and emphasize the importance of transparent criteria and processes for research assessment [51]. It remains to be seen how such initiatives will influence Best Paper awards.”

Below are comments regarding specific lines:

· *73-74**: The authors mention "global calls," which I then expected to be elaborated in the paper, but I found this just in a brief reference on **line 299**. Please make this link clearer.

RESPONSE 18:

To make this link more clear earlier on, we have added a matching statement to one of the sentences in the Introduction:

New lines 128-131: “Studies of elite awards repeatedly reveal limited diversity among the winners, with unsatisfactory progress to a more balanced representation over the years, contributing to calls for more transparency and equity in research recognition [20–22].”

· *87-98**: The claim about the Matthew Effect and historically disadvantages and marginalized groups would be stronger with a reference, if available.

RESPONSE 19:

We have added a few references to strengthen our claim, as suggested:

New lines 95-98: “This feedback loop can reinforce stratification and widen the gaps for historically disadvantaged and marginalized groups in science, such as, women, carers, racial and ethnic minorities, and non-native English speakers [7–10].”

· *90**: Do the authors mean "Studying awards" instead of "Awards"?

RESPONSE 20:

Yes, we meant “Studying awards” – we changed the sentence accordingly:

New lines 99-100: “Studying awards also offers an opportunity to detect and address some of the systemic issues in academia [11].”

· *92**: What "related institutions"? Those giving the awards, e.g., "granting institutions"?

RESPONSE 21:

We changed “related” to “granting”, as suggested:

New lines 100-101: “By examining awards we can gain insights into the culture and values upheld by the granting institutions and organizations and how they are changing.”

· *103**: What do the authors mean by "the ones most valued"?

RESPONSE 22:

We have made this statement more explicit:

New lines 123-125: “Nevertheless, the members of historically underrepresented groups still receive a relatively small proportion of unrestricted research awards, especially the most valued science awards [19].”

· *115**: I would suggest more specific language than "lack of transparency," as I first read this as open-science-related.

RESPONSE 23:

We agree this was unclear. We thus added clarification as to what transparency refers to:

New lines 138-140: “Lack of transparency around the award policies and processes, in particular, can act as an insidious form of gatekeeping, which may inadvertently enable biases via personal preferences and connections [34].”

· *121**: "Prizes" terminology is first introduced here. I had to re-reread since I first thought this was different than awards and instead were what the authors later call "perks." Perhaps establish these as synonyms, possibly even earlier.

RESPONSE 24:

This is a great suggestion. We now establish “award” consistently as a general concept and the word “prize” as when it comes to the cash that comes with the award. :

New lines 148-151: “The landscape of research awards is changing. Research awards are increasingly common across disciplines [3,35], tracking the global growth of the research community [36], the proliferation of learned societies [37], journals [38], and the pursuit of recognition for a broader range of ideas, activities and diverse scholars.”

New lines 333-344:“In terms of the financial benefits, 112 (50%) of the awards descriptions mentioned a monetary prize for the winning authors (Fig 6A and Fig S38). The majority of the cash prizes are less than 2,500 USD (median = 1,086 USD; Fig 6B), but there are also four valued at 10,000 to 25,000 USD (Fig S39). Exploratory text mining revealed award benefits other than cash (Fig S40 and S41).”

New lines 448-451: “Even if the median monetary value of Best Paper awards (1,086 USD) cannot compare with more elite awards [53], they can be a game changer for the early-career, underprivileged and underfunded researchers, especially those from the Global South countries or underfunded labs.”

· *125**: What do the authors mean by "diversity of work"?

RESPONSE 25:

Indeed, this is not the best phrase in this context – we replaced it with a more commonly used expression, “research outputs”.

New lines 151-155: “This pursuit has led to the creation of many types of awards, ranging from those recognizing broad achievements (e.g., the Association for Psychological Science “Rising Star” awards) through to those for specific activities (e.g., communication, mentoring, application) and discrete research outputs (e.g., posters, oral presentations, books, and articles).”

· *134-137**: While I intuitively agree with this claim, I would suggest either citing or soften the language.

RESPONSE 26:

We agree and have modified this sentence to soften the language and provide more details:

New lines 161-164: “While academics may notice who wins such awards within their own disciplines, they may not have a “big picture” of the cross-disciplinary distribution of Best Paper awards – who gets recognised (e.g., in terms of gender and country of affiliation), how (award policies and procedures) and why (award criteria).”

· *138-139**: Are previous studies revealing opaque best paper award criteria limited to reference 33 in computer science? Please reword to clarify.

RESPONSE 27:

We rephrased this sentence to accommodate this comment and added a second example with a reference:

New lines 166-168: “Studies in Computer Science [39] and Ecology and Evolution [11] have revealed opaque award criteria and processes.”

· *146**: What does it mean for an award to "consider equity and inclusion"? Echoing an overarching comment above, do the authors mean whether the research itself is equitable and inclusive, or access to the award? Also, why these specific terms?

RESPONSE 28:

We changed “equity and inclusion” to “equitability of the eligibility and assessment criteria” to make this sentence more explicit and clear.

New lines 194-196: “In Ecology and Evolution, descriptions of awards targeting early- and mid-career researchers seldom consider equitability of the eligibility and assessment criteria; one positive finding was that the proportion of female winners has increased in the last two decades [11]”

· *150**: I was surprised to see that a main aim was to evaluate "gender and affiliation country," given the broader scope presented in the introduction. The authors make this feasibility-based choice clearer later on, but it would have helped me as a reader to already hint at this in the introduction.

RESPONSE 29:

We appreciate this comment. We now expanded our rationale in the preceding paragraph to lead into our aims as follows:

New lines 145-149: “While academics may notice who wins such awards within their own particular disciplines, they do not have a “big picture” of the cross-disciplinary distribution of Best Paper awards – who gets recognised (e.g., in terms of gender and country of affiliation), how (award policies and procedures) and why (award criteria).”

· *156**: Thanks for this great workflow chart! One question towards the bottom: what is "less relevant SA"? Please specificy how this was determined, e.g. in a figure caption.

RESPONSE 30:

To help explain this, we now added in the Figure S1 caption: “When an award was linked to a journal which had more than one SCImago Subject Area (SA) assigned, we used award description to choose the most fitting Subject Area for the award (i.e., we excluded copies of the award record from the less relevant Subject Area – see the bottom right corner of the workflow.”

· *157**: Thanks for sharing the OSF link (<https://osf.io/8mxpq/>) with your protocol. I am confused about the versioning with two files called "BP_awards_protocol_final.pdf": <https://osf.io/93256> created & modified December 26, 2022, and <https://osf.io/vresm> created November 12, 2022 & modified September 17, 2023. It seems the pdf's are both the same and dated 25 December 2022 and one could be removed for clarity.

RESPONSE 31:

We apologise for the confusion regarding the files. The protocol files are versioned and time-stamped for clarity and transparency. We have two protocol versions due to small modifications after feedback from the team members. We have deleted the older file and also changed the reported access link to <https://osf.io/93256>.

· *158**: I believe you searched the top-SCImago-ranked 100 journals OR until 10 awards were found. Please integrate this into the brief methods summary, as it would have helped to understand the subsequent numbers.

RESPONSE 32:

This is a great idea. We incorporated a brief explanation in this sentence, accordingly:

New lines 207-210: “In brief, we first searched for awards associated with the top 100 journals from each of the 27 SCImago Subject Areas (https://www.scimagojr.com/; Fig S2) until 10 potentially eligible awards were found or we reached the 100 journals limit, to achieve a balanced representation across disciplines.”

· *163**: What do the authors mean by "characteristics *representing*..." (emphasis added)?

RESPONSE 33:

We replaced “representing” with “related to”:

New lines 222-223: “Our data extraction covered award characteristics related to transparency, eligibility rules and assessment criteria.”

· *165**: The authors detail how gender was "coded" in the methods and limitations (**lines 375-377**). I would suggest to soften the language here to "we inferred the winning author's gender."

RESPONSE 34:

We changed the wording, as suggested:

New lines 223-226: “For awards specifically highlighting individual article authors (i.e., having a main winner/s rather than the whole authorship team being recognised equally), we inferred the winning author’s gender and country of affiliation, for winners from the years 2001 to 2022.”

· *166-167**: I was confused about the "eligible awards," which though explained later, could be briefly made clearer here.

RESPONSE 35:

To reduce confusion, we removed the first part of this sentence, which now reads as follows:

New lines 226-227: “We present overall results (across all Subject Areas) in the main text and we include equivalent results by Subject Area in the Supplementary Materials.”

· *177-179**: Please clarify why the percentages add to over 100.

RESPONSE 36:

We now added clarification at the end of this sentence as follows:

New lines 235-239: “Of the included awards, 176 (79%) were associated with specific journals (Fig 2A), 144 (65%) with learned societies, and 39 (18%) also mentioned some other organizations being involved (usually journal publishers providing financial or other support: e.g., Elsevier – 15 awards, Wiley – 6; many awards had more than one type of awarding body, so that percentages do not add up to 100%).”

· *179-180**: I am curious what sectors the "other organizations" come from. From the parenthetical, it seems limited to Elsevier and Wiley. The sentence could be clarified.

RESPONSE 37:

We agree that this sentence could be improved. We have modified the wording to clarify that “other organisations” are usually journal publishers:

New lines 235-239: “Of the included awards, 176 (79%) were associated with specific journals (Fig 2A), 144 (65%) with learned societies, and 39 (18%) also mentioned some other organizations being involved (usually journal publishers providing financial or other support: e.g., Elsevier – 15 awards, Wiley – 6; many awards had more than one type of awarding body, so that percentages do not add up to 100%).”

· *182-204**: I found this section challenging to follow. Regarding the header "Awards - equity and transparency," I am unsure why the term "equity" is used. As for "transparency," based on it's earlier introduction, I understand "transparency" to refer to the clarity of award details, which then also covers the subsequent section on assessment criteria. I saw two areas addressed: first, eligibility criteria which includes single/team, career stage, historically underrepresented and marginalized scholars; second, award information which includes assessors, conflicts of interest, (self-)nomination, feedback, contact details. I recognize this section is hard to structure; splitting it up might help.

RESPONSE 38:

We can see the reviewer’s point here. In line with the reviewer’s recommendation, we now split this section into two: “Awards – eligibility and inclusivity” and “Awards – transparency” (New lines 241 and 268).

· *196**: Do the authors mean "reported" rather than "informed"?

RESPONSE 39:

Indeed, this word fits better here – now fixed:

New lines 269-270: “Over half of the awards (114; 54%) reported who the award assessors are (either listing specific names or mentioning journal editors; Fig S8).”

· *200**: Is "process transparency" used to indicate only whether information about nominated articles is provided? This is what I understand from the main text (only one award) and Fig 3A, but Fig S9 has more that 1 "yes" (arts and humanities; nursing). Please clarify this number. If "process transparency" does mean this single practice, the authors might consider removing this extra term and simply specifying the practice for clarity.

RESPONSE 40:

Thank you for spotting this discrepancy! Indeed, there were two awards scored as “yes” here. The first one provided the following statement: “The 9 short-listed papers come from research teams around the globe including Europe, China, the USA and the Middle East. These papers also celebrate the broad range of excellence in nutrition research for which the BJN is well-known including nutritional epidemiological studies, mechanistic and methodological investigations and clinical nutrition studies”. For the second one, all nominees are listed on the award webpage, so anybody interested can create a breakdown according to authors gender or geographic region. When extracting data we used the following rule (from the protocol): “Process transparency – whether breakdown of the applicants / candidates by gender or geographic region is publicly available [*singular variable: yes / no / not available*]”. We have corrected and clarified the sentence accordingly:

New lines 272-274: “Only two awards provided gender or affiliation information about the nominated authors alongside the winning articles (“Process transparency” in Fig 3A; Fig S9).”

· *208**: Might the authors communicate their result with less judgment by removing "not totally opaque," i.e. "assessment criteria that were not restricted to statements..."?

RESPONSE 41:

Following the reviewer’s suggestion, we have softened this sentence:

New lines 285-287: “Most award descriptions were not only brief but also vague – only 40 awards had assessment criteria that were not restricted to generic statements like “excellent research” (19% of awards with descriptions; Fig 3B; Fig S14; [43]). .”

· *220**: It would be interesting to know generally what type of awardee data was available, if any, beyond name (and in part affiliation). For example, was gender ever provided? Affiliation at time of the award?

RESPONSE 42:

Thank you for asking. None of the awardee lists explicitly stated the gender of the winner. Sometimes a profile text was available which included pronouns. We explain in our Methods section that:

New lines 643-645: “We assigned gender to past awardees by using their pronouns, photos, and first names available on award websites, personal, or professional websites.“

Sometimes, affiliation was provided and when missing, we used affiliation listed on the winning article:

New lines 645-646: “We also manually located information on awardees primary institutional affiliations (country and institution) from award websites or awarded articles.”

When extracting data, we categorised the sources of gender information into three categories: name (58%), photo (25%) and pronouns (17%). We note that this does not mean that photo and pronouns were located on the award page. When extracting data, we also categorised the sources of affiliation information into four categories: article (47%), award page (35%), award announcement (15%), and other (3%). We note that such analyses of the sources of gender and affiliation information were not pre-registered for our study and thus we do not report them in the main text, but as part of Supplementary Information (SI_methods_results.html).

· *228**: Was affiliation information retrieved manually or using code?

RESPONSE 43:

We apologise for omitting this detail. We now provide this information in the Methods section:

New lines 645-646: “We also manually located information on awardees primary institutional affiliations (country and institution) from award websites or awarded articles. ”

· *Fig 5**: This data may be better suited to another type of plot, especially given the difference in number of years for the current decade. As I am not a data visualization expert, I unfortunately cannot suggest further. Figs 5C & D have too many layers and colors in the stacked bar chart, making it hard to read. Perhaps a map-based density chart would work.

RESPONSE 44:

We see the reviewer’s point. To accommodate differences in the number of years per decade and numbers of awardees per decade, we use proportions in panels B and D. Country of affiliation data are shown in the same format (stacked bar plot) for consistency and to save space. We would love to present this as map-based density [plots, but this would require fitting three different maps for each decade in each of the panels C and D, while also risking that subtle differences in colour shadings will not be accurately readable to the human eye. Stacked bar plots have an advantage of being compact and exploiting the eyes’ ability to accurately compare lengths of parallel lines, especially when aided with contrasting colours, as used in our plots. Following this reviewer’s suggestion, we now provide colour-shaded map plots as a Supplementary Figure S32 and S33.

· *295**: What are "impact awards" and why could they not be included?

RESPONSE 45:

“Impact awards” are awards that are solely based on simplistic impact metrics, such as numbers of citations or downloads, for example “Most Cited Paper”, “Most Downloaded paper” awards. Since they are, by default, simplistic in their criteria and selection, and usually will not even require an application/nomination, there was no value in including them (but they could be the focus of a separate future project). We reworded this sentence to improve clarity:

New lines 405-416: “Despite the generally poor transparency, we found that 10% of awards descriptions mentioned article impact metrics (counts of citations or downloads), and 4% relied exclusively on such indicators (which is likely an underestimate as we also found some “Impact awards” which are awards based solely on number of citations or downloads, and did not fit our inclusion criteria).”

· *296-297**: The authors might strengthen their critical argument against impact indices with a reference.

RESPONSE 46:

Thank you for this suggestion to strengthen our argument. We have added a reference supporting this statement:

New lines 416-418: “Use of impact indices gives the award criteria a veneer of objectivity and simplifies the assessment process [49].”.

· *309**: Why is "increasingly" in parentheses?

RESPONSE 47:

Thank you for spotting this. “Increasingly” refers to the result presented in Figure 7AB showing that profiles and photos of the winners are now commonly available online in recent years. We simplified the sentence by removing this unnecessary information and replacing other words for consistency.

New lines 434-436: “The visibility comes not only from public award announcements (on websites, editorials, and/or during conferences), but also from invited conference talks, and research profiles and photos published online.”

· *315-316**: That best paper awards "contribute to an invaluable sense of achievement and belonging, especially for minority and underrepresented researchers" is a strong argument and should be cited or made clear that this claim is made on the basis of the presented research (which I don't believe is the case).

RESPONSE 48:

We agree that this statement cannot be derived from our research. Instead, we now provide a reference from the research literature that supports this statement.

New lines 452-454: “They not only provide a financial boost, but also demonstrate a record of attracting external funding and contribute to an invaluable sense of achievement and belonging, especially for minority and underrepresented researchers [54].”

· *317**: As I wrote above, this paragraph does not seem to focus on "equitability and inclusiveness." I think this important argument and reflection could be strengthened by restructuring this paragraph and the subsequent one.

RESPONSE 49:

We have made adjustments to this and the following paragraph (new lines 456-489), following suggestions from all reviewers to clarify and strengthen our arguments.

· *333-346**: I applaud the authors' attempt to provide more actionable recommendations based on their research, and appreciated their used of an established framework (thank you for introducing me to the SPACE rubric). However, as this framework is extensive and rather complex, I wonder if it could be accompanied by a concrete example (e.g., in a "Box" in the article) of a specific institution granting a best paper award, perhaps one that is further along in the reform. If feasible, this would help not only academic readers, but also journal leadership looking to apply this framework to reform their journal's awards. A few comments within the table:

RESPONSE 50:

Unfortunately, we did not find any institution yet that would fulfill all or even the majority of the recommendations. We considered adding examples as a box, but it would be impossible to cover all the recommendations in the limited space (e.g., providing examples of full winner’s profiles in multiple languages and with photos) and also, we would be at risk of breaching copyright laws if reusing materials posted on journal and learned societies’ websites. We could consider using links to existing online award descriptions, but it is also risky given that such links can be changed by the website owners and are likely to become defunct rapidly. Finally, we generally do not think there is a “one fits all” solution and each awarding body would need to decide how to implement our recommendations for their specific circumstances.

· **Standards for scholarship**: Perhaps list a few "buzzwords" so that readers looking at only this table can follow. The last two bullets seem to go beyond the "foundation" column, and could be reworded to focus on the hard work of established shared understanding.

RESPONSE 51:

We added examples of “buzzwords”, as suggested:

Table 1:

-	“Clearly define what “Best” stands for, without relying on buzzwords (e.g., excellence, novelty, significance).”

We also softened the last two recommendation points in this category as follows:

Table 1:

-	“Focus on increasing equity and support for traditionally underrepresented and minoritized groups.”

-	“Work on diagnosing the nature of inequities that exist in the current (or traditional) policies and procedures.”

· **Process mechanics and policies**: Again "ensure" sounds like it goes beyond "foundation." What is meant by "the narrow circle"?

RESPONSE 52:

Since it is our first recommendation in this category, we changed “ensure” to “foster”:

Table 1: “Foster assessment of nominations that is consistent and structured to minimize potential biases.”

We also clarified the last recommendation in this category:

Table 1: “Value diverse and inclusive research teams (e.g., collaborating outside the immediate circle of colleagues, gender-balanced teams).

· *349**: Recommendations to researchers seem to revolve around asking/communicating with the awarding body; perhaps this could be more informative in the paragraph's topic sentence that "straightforward."

RESPONSE 53:

We have made this sentence more informative as follows:

New lines 514-515: “Recommendations for individual researchers potentially applying or nominating for the awards are focused on proactive communication with the awarding bodies.”

· *377-378**: The authors mention one additional dimension of researcher diversity which they considered and decided not to include. If other dimensions were considered, such as those in **lines 396-397**, please reference them. If not, please elaborate on how the dimensions were selected, first, for consideration, and, second, for analysis.

RESPONSE 54:

Following this valuable suggestion, we elaborated as follows:

New lines 552-554: “Other dimensions of diversity that cannot be surveyed from publicly available information include disability, linguistic, socio-economic, educational background, etc.”

· *380-382**: This claim would be strengthened by a reference.

RESPONSE 55:

We have added a reference for this statement:

New lines 555-557: “Nonetheless, affiliation information tends to reflect the prestige and resources available to the researcher during their research work and how the world sees research success of a country [60].”

· *440-441**: How did you select "awards that carry the most prestige"? Is this by SCImago ranking?

RESPONSE 56:

Yes, it is based on SCImago ranking. We now explain this in the following sentence:

New lines 618-620: “To collate such a sample of awards, we conducted a systematic-like search based on top-ranking journals, and associated learned societies, based on the assumption that awards associated with highly ranked journals will be more prestigious.”

· *447-449**: On my first reading, it was unclear whether the "10 potentially relevant awards" were per journal or field. This might be clarified by rephrasing the sentence to leading with "We stopped screening each Subject Area...".

RESPONSE 57:

Thank you for bringing our attention to this. We have clarified that we mean 10 per Subject Area.

New lines 625-628: “We stopped screening when we located 10 potentially relevant awards per Subject Area or when we reached the threshold of 100 screened journals per Subject Area.”

· *468-469**: Are the award description pdfs made available? I had trouble finding them in <https://zenodo.org/doi/10.5281/zenodo.10056492>

RESPONSE 58:

The archived award descriptions are publicly available at <https://osf.io/yzr7a/>. They are compressed into a single file (awards_pages_pdf.zip).

· *507-509**: Kudos to the authors for the open data and code. The OSF at <https://osf.io/yzr7a/> seems to be private and should be made accessible. The GitHub repo has an extensive readme, which is helpful for orientation. However, I imagine I would struggle to reuse the data and would suggest adding a data readme, list of datafiles/csvs, and a codebook. Recognizing that this project produced many intermediate files, I suggest more detailed documentation at least of the final version of the data used for the analysis and claims presented in the paper; this database of awards and winners, complimented by the research team's manual coding, could be useful to future potential award applicants and meta-researchers alike. Please note that I recognise the time-consuming nature and pain of data documentation and thank the authors in advance for this extra effort for the sake of reusability.

RESPONSE 59:

We apologise for the inconvenience. We forgot to switch this OSF repository to public mode. It is public now. Thank you for the excellent suggestion regarding the GitHub repository. We added the list and brief descriptions of key data and code files to the README.md, as suggested under the “Data and code files” section, as follows:

README.md lines 76-91:

“

Data:

- *BP_awards_lists_SHAREDCOPY - indiv_winners_20230915.csv* - data on the extracted characteristics of past individual award winners (focus on gender, and country of affiliation). Meta-data is provided in a separated .csv file.

- *BP_awards_lists_SHAREDCOPY - indiv_winners_meta-data.csv* - meta-data file for the data on individual winners (BP_awards_lists_SHAREDCOPY - indiv_winners_20230915.csv).

- *BP_awards_lists_SHAREDCOPY - people_tasks log.csv* - record of the researcher contributions to the project. First columns includes researcher names and the last two columns show the authorship order and position, respectively. Numerical columns with "(SA)" in the name represent counts of Subject Areas researcher contributed to, e.g., by screening or extracting award data. All other numerical columns are binary variables, where 1 stands for "yes" (i.e., "contributed""), and 0 stands for "no" (i.e., "not contributed").

- *BP_awards_lists_SHAREDCOPY - scimagojr_2021_SA.csv* - list of 27 SCImago Subject Areas (SA) with progress and contributions records. The records include dates and names of contributors for different stages of the project workflow.

- *duplicated_main_extractions.csv* - a subset of extracted award characteristics where data was extracted by at least two independent researchers. Meta-data is the same as for the main dataset provided in the file Survey-Best_paper_awards (Responses)_SHAREDCOPY_checked.xlsx.

- *scimagojr 2021 Subject Areas_screening.xlsx* - lists of journals for each of 27 SCImago Subject Areas (SA) with associated SCimago 2021 data and records of screening decisions and comments. Each SA is provided in a separate Excel tab, with the first tab providing meta-data.

- *scimagojr country rank 2021.csv* - SCImago Country Rank data for 2021 downloaded from https://www.scimagojr.com/countryrank.php. The list of 235 countries with their data on: SCImago Rank, Country	Region,	Documents,	Citable documents,	Citations,	Self-citations,	Citations per document,	H index. For information on the SCImago data sources see https://www.scimagojr.com/help.php?q=FAQ.

- *Survey-Best_paper_awards (Responses)_SHAREDCOPY_checked.xlsx* - raw data extracted via Google Form and saved as a spreadsheet. The extracted data has been cross-checked and supplemented with comments, corrections and final inclusion decisions. Meta-data is provided in the second tab of the file.

R Code:

- *SI_methods_results.Rmd* - R code and markdown file with all analyses and visualisations.

- *SI_methods_results.html* - knitted code and results html file for the website.

“

All meta-data are also available in the html SI file as Tables S1-S4.

To reiterate, this paper makes a valuable addition to the meta-research literature, and I believe the outstanding issues could be addressed in a revision. I ask the editor to please note the limitations of my review due to time constraints; I could not extensively review the supplement, protocol, code, or data.

RESPONSE 60:

Thank you for your time spent on reviewing our work.

Best regards,

Maia Salholz-Hillel

## **Reviewer #3:**

[identifies herself as Tracey Weissgerber]

The authors systematically assessed criteria and characteristics for best paper awards in a multifield sample (n = 222 awards from 27 fields), as well as demographic characteristics of past winners. They observed a lack of transparency surrounding criteria on which papers were assessed and details of the assessors and assessment procedures. Available information suggested that awards prioritized "excellence", and rarely mentioned reproducible research or open science practices.

RESPONSE 61:

Thank you for your accurate summary of our work, as well as for overall positive and valuable feedback on our study.

Strengths of the study include a pre-registered protocol, and available data and code. A major limitation is the sampling frame, which focused on creating a snapshot of the first 4-10 high prestige awards identified from each field, rather than providing a more comprehensive assessment of systematically identified best paper awards across fields. Awards targeting specific career stages were included, whereas awards targeting underrepresented groups were excluded. The authors offer extensive recommendations based on their findings. While the recommendations may be quite valuable, the authors may wish to soften the language surrounding these recommendations given the study limitations. The writing quality is generally good, however, key points can be difficult to find amidst extra words and details.

RESPONSE 62:

We appreciate this insightful feedback. Following this feedback and more specific comments listed below, we softened the language in several places and improved the clarity of our writing (please see our responses below for specifics).

Introduction

1. The introduction is very long and feels like a literature review. The study goal is lost amongst the details. Offer a concise overview of things that are essential to understand the study rationale.

RESPONSE 63:

We appreciate your comment. We managed to shorten this section while still providing all necessary background and study aims. Our Introduction covers a brief introduction to the significance of studying awards, their relationship to Open Science practices and to EDI, which are all relevant to the data we collected via our survey. We believe that any shorter would not allow us to provide sufficient rationale for our research that is grounded in the existing literature.

Deleted sentence: “By systematically collecting data on awards processes and policies we could not only fill the knowledge gap but also contribute to the movement towards more equitable and accountable awards.”

Please note that there are also other small deleted text fragments in this section (please refer to the version with tracked changes).

2. The second clause of the third sentence either needs cited evidence, or should be softened considerably ("awards…open doors to jobs, promotions and funding"). This mirrors a similar statement in the abstract. Similar claims are made without citation in the discussion (lines 312-317). If this is the authors' opinion and evidence is not yet available, clearly state this.

RESPONSE 64:

Following the reviewer’s suggestion, we softened the sentence in question and added references in both the Introduction and Discussion.

New lines 90-92: “Awards can feature prominently on individual curriculum vitae (CVs) and grant applications, opening doors to jobs, promotions, and funding [2,3].”

New lines 446-457: “They not only provide a financial boost, but also demonstrate a record of attracting external funding and contribute to an invaluable sense of achievement and belonging, especially for minority and underrepresented researchers [54].”

Methods

1. I may have missed it, but I could not find details on how assessors were trained or whether they had to meet specific performance criteria to perform assessments.

RESPONSE 65:

This is a great point. The team members contributed to study design and provided feedback on the study protocol. We then used pre-piloted Google Forms when extracting data from award descriptions and provided all assessors with detailed instructions, including examples. There were some overlaps in awards between Subject Areas, and we also had 14 awards that were independently extracted by two to four team members (please see “duplicated_main_extractions.csv”), which were used to further check consistency of extractions. To clarify our approach, we now added a brief explanation in the Methods section:

New lines 650-652: “We followed an agreed protocol and instructions for screening and data extraction process based on custom-built Google forms to assure high consistency of all extracted data.”

2. The second assessment appears to be for verification, rather than an independent assessment. The authors may wish to acknowledge this as a limitation.

RESPONSE 66:

We stated in our Methods that the extracted data was “independently cross-checked by a second reviewer”. To make the sentence in question less ambiguous, we removed the word “independently” from the sentence, which now reads:

New lines 652-653: “Extracted data were cross-checked by a second reviewer.”

We assume that by “independent assessment” the reviewer has meant independent extraction, which we did not do for the full data set. Given the amount of work involved in data extraction it was not feasible to do independent double-extraction of all the data. However, cross-checking of data is a second-best practice and assures good data quality. There were some overlaps in awards between Subject Areas, and we also had 14 awards that were independently extracted by two to four team members (see “duplicated_main_extractions.csv”), which were used to further check consistency of extractions.

3. The study sample covers a 22-year period, however, currency conversion for cash prizes was based on 2023 exchange rates. The analysis does not appear to account for inflation. Were only 2022 amounts considered?

RESPONSE 67:

This is a very insightful comment. We extracted data on award monetary values in 2023 and thus we assumed that these values are current for the year 2023. We do not have any data or draw any inferences on the monetary values of the awards in earlier years.

Results

1. In Figure 2, it is not clear how career stages were defined. Order the career stages logically, rather than alphabetically.

RESPONSE 68:

Thank you for this comment – the career stages (and other values in “upset plots”) are ordered automatically by frequency. Changing them to chronological order would disrupt the upper part of the graph making it less readable. We now added a clarification to the figure legend as follows:

“Fig 2. Summary of included awards.

(A) Awarding bodies. (B) Award target researcher career stage. “Not available” stands for cases with no award description. The dark-colored dots in the bottom part of the graphs indicate which combination of the Awarding bodies (A) or target career stages (B) a given vertical bar above represents (e.g., first bar in panel A shows the count of awards that are associated with both a specific journal and a learned society, and the second bar is for awards associated with a journal only). Awarding bodies and target career stages are shown in the order of their decreasing frequency.”

2. In Figure 3, 6 and 7, the colors and shading are distracting. Solid colors, using a colorblind accessible color palette, might be more effective. The order of colors in the legend should match the order in the figure - the order is currently reversed.

RESPONSE 69:

We included the patterns to make this figure more colourblind accessible. However, we are happy to remove these patterns given that we have also already used a colourblind friendly palette in all of our plots (https://github.com/cran/wacolors).

3. In Figure 4, rigor and rigour could easily be combined, as these are simply different spellings.

RESPONSE 70:

Thank you for this great suggestion. We now fixed this in Figure 4.

Discussion

1. The discussion is quite long. Avoid duplication. Offer a more concise and focused presentation to make key points accessible to readers.

RESPONSE 71:

We agree that the discussion is quite long (seven pages). However, a large part is dedicated to study limitations, recommendations (most of them are already placed in Table 1), and future directions, which are all crucial to this work. The actual discussion of the survey results takes only 3.2 pages, which is not long and is firmly centred around our key findings, which we only briefly reiterate. We have struggled to make this section shorter without making it superficial.

2. In the recommendations section, the authors offer extensive recommendations for organizations that offer awards, followed by brief recommendations for individuals who are applying or being nominated; then suggest that the broader research community should reconsider whether to use these awards in researcher assessments. One could consider placing the last point first, and reframing it to include what criteria best paper awards should meet, if these awards are going to be used in research assessment. This may help to frame the recommendations for organizations that offer awards and those who are applying or nominating researchers for awards. Statements about the current use and/or impact of best paper awards in research assessment need to be supported by evidence, as noted previously.

RESPONSE 72:

We now moved the recommendations for the broader researcher community before recommendations for the awarding bodies, as suggested. This paragraph includes a specific recommendation “increasing transparency of the awards would be a first step toward building community trust, revealing their true value and acknowledging limitations of Best Paper awards.“ We have amended the legend of Table 1 to indicate that these recommendations also would be helpful for having more transparency when including such awards in research assessment. We also added several references to support the statements included in the Discussion, as suggested.

Table 1.:

“Recommendations for awarding bodies establishing new and managing existing Best Paper awards across disciplines. These recommendations could also be helpful for improving transparency and equity when considering including Best Paper awards in research assessment. The table is based on SPACE rubric [57,58], but the specific recommendations are largely derived from the award survey results.”

3. Thank you for including a limitations section. While awards were systematically assessed, the sampling frame used to identify awards is not particularly systematic. The authors note this as their first limitation (the statement about creating a "snapshot" of best paper awards across fields is a nice description), however, the reasons why this is important aren't fully elaborated. Many of the decisions made by the authors when setting inclusion criteria and screening potential awards for inclusion may have impacted the final sample (e.g., focusing on the oldest or most important awards, excluding awards targeted towards underrepresented groups, stopping after 10 awards were included for a particular field). The potential impact of such decisions on generalizability should be noted. The per field sample size is quite small (4-10 papers) and selection of different awards could have resulted in different characteristics across the sample. The first paragraph of the results does address some elements of sample selection, however some of the most important items are missing (see examples listed above). A clear concise statement about features of the sample that may impact generalizability should be included in the first paragraph of the results - it would be nice to see the "snapshot" language used before the limitations section. Given these potential limitations to generalizability, the authors may wish to soften some of the language surrounding their recommendations.

RESPONSE 73:

Following the reviewer’s recommendations, we have added a statement about features of the sample that may impact generalizability in the first paragraph of the Results and we have expanded the limitations section to include potential limitations to generalizability due to our study design.

New lines 213-214: “We acknowledge that the resulting snapshot of Best Paper awards may not be fully generalizable due to our search procedure and selection criteria (see Discussion).”

New lines 523-525: “First, our process for identifying relevant awards for inclusion was stratified and thus not exhaustively comprehensive or fully random which may reduce the generalizability of our findings.”

4. The authors note that "Noticing, valuing and promoting the work by the researchers from the Global South…would require changes in how Best Paper awards are defined, managed and presented." This requires a brief explanation. Would the authors recommend targeted awards, or changes in the way that non-targeted awards are advertised or given? Caution is needed here to avoid speculation, as awards targeting underrepresented groups were excluded from the sample.

RESPONSE 74:

Thank you for this comment – we agree that it requires further explanation. The following paragraph introduces our more elaborated recommendations and Table 1 includes a list of specific recommendations many of which are related to EDI. These recommendations focus on changes to existing awards rather than creating targeted awards – as reviewer correctly notes our sample did not include targeted awards and, thus, we do not have evidence to provide recommendation to this category of awards. To reduce confusion, we have modified this sentence as:

New lines 470-473: “Noticing, valuing, and promoting the work by the researchers from the Global South (as well as other underrepresented groups, such as women) would require changes in how Best Paper awards are defined, managed, and presented, as outlined below.”

5. In line 356, the authors refer to the broader scientific community. As humanities and social sciences are included in the sample, perhaps "research community" would be more inclusive.

RESPONSE 75:

Thank you, we have changed this as suggested:

New lines 484-485: “Recommendations for the research community include asking whether we should use Best Paper awards in researcher assessments.”
